# Fetal Alcohol Syndrome and Essential Fatty Acids

**DOI:** 10.1371/journal.pmed.0030247

**Published:** 2006-05-30

**Authors:** Undurti N Das

**Affiliations:** **1**UND Life SciencesShaker Heights, OhioUnited States of America

Essential fatty acids (EFAs) are structural components of the brain and influence nerve conduction and transmitter release and action [ 1]. Prostaglandins (PGs) derived from EFAs and polyunsaturated fatty acids (PUFAs) modify nerve conduction and transmitter function. Ethanol (1) reduces blood linoleic acid (LA) levels; (2) blocks conversion of LA and gamma-linolenic acid (GLA) to arachidonic acid (AA), as well as alpha-linolenic acid (ALA) to eicosapentaenoic and docosahexaenoic acids (EPA and DHA, respectively), by inhibiting delta-6 and delta-5 desaturases; and (3) enhances the conversion of dihomo-gamma-linolenic acid (DGLA) to PGE1 [ 1].

Ethanol-enhanced interleukin-6 (IL-6) and tumor necrosis factor-alpha (TNF-alpha) production suggest that immunological mechanisms play a role in ethanol-induced diseases. Cerebral cortex from chronic ethanol-fed rats showed up-regulation of inducible nitric oxide (iNOS), cyclo-oxygenase-2 (COX-2), IL-1-beta, and activation of transcription factors nuclear factor-kappa B (NF-kappa B) and AP-1—effects that increased both caspase-3 and apoptosis [ 2].

Brains of ethanol-treated mice show raised phospholipase-A2 and phospholipase-C activity [ 3] that can cause the release of AA, EPA, and DHA from membrane phospholipids. AA, EPA, and DHA form precursors to eicosanoids—lipoxins (LXs), resolvins, and neuroprotectin D1 (NPD1) [ 4]. LXs, resolvins, and NPD1 suppress IL-6 and TNF-alpha production. PGs, LXs, resolvins, and NPD1 precursor supplies are limited; therefore, when ethanol consumption is substantial, tissue concentrations of PUFAs decline, leading to a fall in the synthesis of various PGs, LXs, resolvins, and NPD1.

DHA and other PUFAs released from the membrane in response to neurotransmitters [ 5] activate retinoid X receptor (RXR) as PUFAs form ligands for the RXR [ 6]. DHA deficiency results in impaired spatial learning and other abnormalities [ 7]. RXR heterodimerization partners peroxisome proliferator-activated receptors (PPARs), liver X receptors, and farnesoid X receptors that are essential for regulating energy and nutritional homeostasis. PUFAs, being ligands for RXR and PPARs, modulate these and other regulatory events by binding to these nuclear receptor heterodimers.

AA, DHA, EPA, LXs, resolvins, and NPD1 have neuroprotective actions [ 4]; inhibit IL-6 and TNF-alpha production that is neurotoxic; and increase synthesis of endothelial nitric oxide (eNO), a neurotransmitter. When neurons are depleted of their PUFA content, as happens after high alcohol intake, formation of LXs, resolvins, and NPD1 will be suboptimal and, hence, neuronal death induced by TNF-alpha cannot be antagonized. This results in fetal alcohol syndrome (FAS).

Nicotinamide is necessary for delta-6 desaturase and enhances the formation of various PUFAs and their products [ 8], suggesting that the neuroprotective action of nicotinamide against ethanol-induced FAS, as described by Alessandro Ieraci and Daniel Herrera [ 9], could be attributed to its action on these lipid pathways.
